# Progress in Medicine

**Published:** 1898-03-12

**Authors:** 


### Progress in Medicine.

### DISEASES OF THE BLOOD.

**Leukæmia.**—Van der Wey[1] has been able to estimate the ingestion and excretion of nitrogen in this disease in two cases. The first, a young man of 23, showed an excess of excretion in the urine and fæces over the nitrogen taken in food, for respective periods of ten, eight, nine, and six days, of 19·5 grammes, 8 grammes, 17·8, and 18 grammes, but the second patient, a girl of 19, showed a gain of 2 grammes in a week. The greatest amount of uric acid was excreted during febrile states, but even in apyrexia there was more than normal excretion. He appears to believe that acute and chronic leukæmia are really the same disease, since he found during a certain stage of the chronic disease the same histological and other symptoms as in Fränkel's acute leukæmia. A case of lymphatic leukæmia is discussed by W. F. Edwards,[2] where the leucocytes were in the proportion of one to eight red corpuscles, the increase being almost entirely due to the lymphocytes. No myelocytes or nucleated red cells were to be seen, nor the ordinary hyaline cells, while the granular eosinophile cells were few in number. Later on, the leucocytes nearly equalled the red corpuscles, and the patient died after six weeks' illness. There were no hæmorrhages, but the glands in various parts of the body were enlarged, and the spleen among others. This change in the spleen is rarely seen to any extent in the lymphatic form of the disease, but in other respects the case is a fairly typical one. The writer comments on the close relation to acute forms of Hodgkins' disease where leucocytosis has occurred, and thinks that possibly both these diseases and sarcoma are manifestations of the same disease. With reference to the distinction between leucocytosis and leukæmia, Buchanan[3] lays down that the latter is more permanent and shows abnormal cell forms, such as myelocytes (a few of which were to be seen towards the end of Edwards' case). He considers, moreover, that between the two forms of leukæmia, lymphatic and spleno-myelogenous, are found mixed varieties, and probably there is no radical distinction between them. The important thing is that abnormal elements remain present in spite of treatment, and that relapse invariably occurs. Hence the analogy to malignant growths. On the other hand, in Hodgkins' disease there seems to be some connection with tuberculosis. C. H. Lewis[4] refers to the tuberculous symptoms seen in Hodgkins' original cases and in the early ones described by Wilks, and mentions one of Duplay's in which both diseases were clearly present. In a patient of his own he gives evidence of a similar association. The symptoms commenced during pregnancy, with enlargement of the glands in the neck; similar changes took place elsewhere, and a cough developed after her confinement. Apical phthisis, with bacilli in the sputa, some diminution of red corpuscles, and no marked leucocytosis were observed, and death took place from exhaustion. The post-mortem showed enlargement of the spleen and abdominal glands, which formed large masses. This was due to a great proliferation of round cells and much new connective tissue. It is possible that some of the cervical glands were tuberculous, as some showed fluctuation, but those elsewhere remained unchanged up to death.

The blood conditions in malignant disease are especially important where there are no localising signs of the new growth. Thus, in the diagnosis of thoracic tumour from aneurism, an examination of the blood may be of great service. Sailer and A. E. Taylor[5] give an analysis of the blood in 22 cases of advanced cancer. The average of hæmoglobin was 35 per cent., which is lower than the 45 to 65 percentage generally described. It would seem, however, that the loss of corpuscles is usually less than the loss of hæmatin. The red cells averaged 3,085,000, poikilocytosis was very variable, nucleated red cells were seen in nearly half of the cases, and leucocytosis in 15 out of the 22 cases. Indeed, this is generally absent in cancer of the œsophagus with starvation, and in cancer on the face or extremities. The differential count showed generally an increase in the mononuclear and transitional forms over the lymphocytes, and this would seem to be important in the diagnosis from pernicious anæmia. The paper concludes with a useful bibliography and an account of the chemical studies of the blood recorded by others. Generally it has been said that the specific gravity is diminished like the amount of the dried residue. Probably, too, the organic and inorganic acids are increased, and the alkalinity diminished.

**Bacteria in the Blood.**—An interesting investigation bearing on the bactericidal power of blood has been made in the Johns Hopkins' Laboratory.[6] The observers find that the granules in the blood plasma, which resemble those seen in the eosinophile leucocytes, are of importance, though it is exceedingly difficult to separate them from the leucocytes floating near them. The "clumping" power in typhoid serum ceases if the granules and leucocytes are removed, and the authors think that eosinophile cells give up or set free their granules, which attack the bacteria. Indeed, they have been able to observe "clumped" bacilli surrounded by actively-moving granules. Their work agrees with the observations of Kanthack and Hardy, who found that the destruction of anthrax in the frog was commenced by leucocytes, which discharged streams of granules among the bacilli. The latter showed signs of degeneration, and then hyaline non-granular cells approached and took up into themselves the helpless bacilli. If this theory be correct, we see that cell-free fluids can destroy bacteria by their granules, while it is still true that phagocytosis by some of the leucocytes takes place after the bacteria are destroyed by the granular poisons. In connection with the activity of the eosinophile (and possibly the neutrophile) granular cells in this direction, it is worth noticing that Thayer[7] found an unheard-of percentage of eosinophiles in two cases of trichinosis; these cells even exceeded 68 per cent. of the whole number, with a marked decrease in the neutrophiles. Whether this is diagnostic of trichinosis remains to be seen.

Though the discovery of bacteria in the blood is too uncertain to be of much value for the diagnosis of the disease, a positive result shows that more than a local infection has occurred, and that a general septicæmia has taken place. Kohn's[8] results in pneumonia are the most interesting of the numerous investigations he made. Out of 32 cases of pneumonia he found the diplococcus in the blood in nine, seven of which were fatal, and the other two presented secondary abscesses; while, on the other hand, no death from pure pneumonia took place in the patients whose blood was free from the coccus. He therefore looks on the appearance of cocci in the blood as of the worst augury, and as generally indicating a septicæmic condition or multiple local centres of the poison. Since blood serum has been so often tested as a means of diagnosis, those cases where it is cloudy or turbid have acquired additional interest. Castaigne[9] finds that the turbidity is due (1) very rarely to fatty particles, (2) more usually to the presence of a quantity of molecular albumen. It may occur in perfect health, especially after a meal, or it may be found in conjunction with nephritis, or during an acute disease such as typhoid. Further investigations showed that the associated nephritis is always an epithelial one, and the appearance of turbidity is so far a proof that no interstitial or arterial changes have taken place in the kidneys.

**Chlorosis.**—A severe blow has been given to a once accepted theory of this disease—Virchow's narrow aorta—although it has long ceased to occupy the place which it once did. Suter[10] has measured the aorta in 2,719 cases, and finds no relation between its size and any forms of anæmia; while the aorta varies in size with age, sex, and size of the individual. He finds that there is no such thing as a narrow aorta in the sense of Virchow and Rokitansky, nor is it true that those who die of puerperal fever, phthisis, or carcinoma have specially narrow or wide aortas. Biernaki[11] points out that the most constant change in the blood in chlorosis is hydræmia, or deficiency of albuminoid bodies. The colour of the skin has been thought to be due to a deficiency of hæmoglobin, but the writer finds that there is no relation between the two changes. Similarly the dyspnœa of the disease has been attributed to the deficiency of oxygen in the scanty hæmoglobin present; but, as a matter of fact, there may be more oxygen present than in normal blood. Gilbert[12] defended at Moscow the old theory of arterial hyperplasia as a cause of chlorosis. Among the other effects of this hyperplasia the small aorta is found, but the writer does not go so far as to say that all cases of chlorosis depend on this cause; and, indeed, he acknowledges that similar changes in the aorta may occur without chlorosis making its appearance. Dr. Sansom still holds Virchow's doctrine that chlorosis may depend on an imperfectly developed aorta, and thinks that the smallness of the aorta may often be due to a similar imperfection in the pulmonary artery. The latter affection is one eminently capalbe of cure by baths, tonics, and exercises for increasing the capacity of the lungs.

[1] Internat. Med. Mag., June. [2] Med. Chronic., July. [3] Int. Med. Mag., Nov. [4] Med. Rev., Aug. 28. [5] Int. Med. Mag., July. [6] Johns Hopkins Bulletin, No. 81, 1897. [7] Lancet, Sept. 25. [8] Amer. J. Med. Sc., Oct. [9] B.M.J., Sept. 18. [10] Amer. J. Med. Sc., Nov. [11] B.M.J., July 24. [12] Med. Rec., Oct. 2.

